# The Dutch Multidisciplinary Occupational Health Guideline to Enhance Work Participation Among Low Back Pain and Lumbosacral Radicular Syndrome Patients

**DOI:** 10.1007/s10926-021-09993-4

**Published:** 2021-07-27

**Authors:** J. W. H. Luites, P. P. F. M. Kuijer, C. T. J. Hulshof, R. Kok, M. W. Langendam, T. Oosterhuis, J. R. Anema, V. P. Lapré-Utama, C. P. J. Everaert, H. Wind, R. J. E. M. Smeets, Y. van Zaanen, E. A. Hoebink, L. Voogt, W. de Hoop, D. H. Boerman, J. L. Hoving

**Affiliations:** 1 Department of Public and Occupational Health, Amsterdam UMC, Amsterdam, The Netherlands; 2 Netherlands Society of Occupational Medicine (NVAB), Utrecht, The Netherlands; 3 Dutch Society of Insurance Medicine (NVVG), Amsterdam, The Netherlands; 4 Department of Epidemiology and Data Science, Amsterdam UMC, University of Amsterdam, Amsterdam Institute of Public Health, Amsterdam, The Netherlands; 5 Research Center for Insurance Medicine (KCVG), Amsterdam, The Netherlands; 6 Dutch Association of Medical Officers in Private Insurances (GAV), Utrecht, The Netherlands; 7 Arbo Unie, Arnhem, The Netherlands; 8 Department of Rehabilitation Medicine, Maastricht University, Maastricht, the Netherlands; 9 Libra Rehabilitation and Audiology, Eindhoven, The Netherlands; 10 Netherlands Society of Physical and Rehabilitation Medicine (VRA), Utrecht, The Netherlands; 11 Dutch Association of Physiotherapists Working in Occupational Health and Ergonomics (NVBF-KNGF), Amersfoort, The Netherlands; 12 Department of Orthopaedic Surgery, Foundation for Orthopaedic Research Care and Education (FORCE), Amphia Hospital, Breda, The Netherlands; 13 Dutch Association for Patients With Back Problems (NVvR), Rotterdam, The Netherlands; 14 Dutch Association for Labour Experts (NVvA), Nijkerk, The Netherlands; 15 Department of Neurology, Rijnstate Hospital, Arnhem, The Netherlands; 16 Netherlands Society for Neurology (NVN), Utrecht, The Netherlands

**Keywords:** Occupational health, Guideline, Low back pain, Sciatica, Therapy

## Abstract

*Purpose* Based on current scientific evidence and best practice, the first Dutch multidisciplinary practice guideline for occupational health professionals was developed to stimulate prevention and enhance work participation in patients with low back pain (LBP) and lumbosacral radicular syndrome (LRS). *Methods* A multidisciplinary working group with health care professionals, a patient representative and researchers developed the recommendations after systematic review of evidence about (1) Risk factors, (2) Prevention, (3) Prognostic factors and (4) Interventions. Certainty of the evidence was rated with GRADE and the Evidence to Decision (EtD) framework was used to formulate recommendations. High or moderate certainty resulted in a recommendation “*to advise”*, low to very low in a recommendation “*to consider”*, unless other factors in the framework decided differently. *Results* An inventory of risk factors should be considered and an assessment of prognostic factors is advised. For prevention, physical exercises and education are advised, besides application of the evidence-based practical guidelines “lifting” and “whole body vibration”. The stepped-care approach to enhance work participation starts with the advice to stay active, facilitated by informing the worker, reducing workload, an action plan and a time-contingent increase of work participation for a defined amount of hours and tasks. If work participation has not improved within 6 weeks, additional treatments should be considered based on the present risk and prognostic factors: (1) physiotherapy or exercise therapy; (2) an intensive workplace-oriented program; or (3) cognitive behavioural therapy. After 12 weeks, multi-disciplinary (occupational) rehabilitation therapy need to be considered. *Conclusions* Based on systematic reviews and expert consensus, the good practice recommendations in this guideline focus on enhancing work participation among workers with LBP and LRS using a stepped-care approach to complement existing guidelines focusing on recovery and daily functioning.

**Supplementary Information:**

The online version contains supplementary material available at 10.1007/s10926-021-09993-4.

## Introduction

Low back pain (LBP) is a major health problem in the world, and is also the number one cause of disability globally [1]. It is one of most common reasons why people consult a doctor, experience activity limitations or miss days at work [2]. Disability is highest in working age groups [1]. LBP is a complex condition with pain and disability aspects in different manifestations, influenced by various biological, psychological and social factors. In most cases a specific cause cannot be established, referred to as non-specific LBP [3]. Patients diagnosed with lumbosacral radicular syndrome (LRS) experience radiating pain variating from dull, aching and difficult to localize, to sharp and burning in buttock and/or the leg, which could be accompanied by one or more other symptoms suggestive for radiculopathy, such as sensory symptoms, muscle weakness or other abnormalities in neurological examinations. LRS is often caused by irritation/inflammation of the nerve root, less often by direct pressure from a herniated disc.

LBP is the most common cause of medically certified sick leave and early retirement. The onset can be work-related [4, 5], many workers with long-lasting work absenteeism attribute their LBP to work [6]. However, current guidelines managing LBP and LRS focus mainly on pain relief and recovery of daily functioning in the general population [7–9]. As far as we are aware of, a multidisciplinary guideline with the focus on effective interventions for work participation is currently not available. In this paper, we present the main results of the Dutch multidisciplinary Occupational Health Guideline to enhance work participation among workers^1^ with LBP and LRS. With its main focus on effective intervention strategies for work participation through work related outcomes, such as return to work and days of sick leave, the guideline complements existing recommendations to improve daily functioning. Besides that, it contains detailed tables with risk factors and prognostic factors specified for workers, as well as interventions for the prevention of LBP and LRS in work. This guideline is developed for occupational physicians (OP) and insurance physicians (IP), operating in the social or private sector, who manage work participation on workers with LBP and LRS. Other occupational health (OH) care professionals, like occupational hygienists, ergonomists, occupational physiotherapists, nurses and labour experts or occupational assessors, can also use this guideline in their work, as well as health care professionals like general practitioners (GP) and physiotherapists.

In the recommendations four topics, determined in consultation with stakeholders relevant to the occupational field, are addressed: 1) risk factors associated with a new episode or recurrence of LBP and LRS in workers resulting in reduced work participation; 2) interventions preventing development of LBP and LRS in work; 3) prognostic factors for reduced work participation in workers with LBP and LRS; and 4) interventions maintaining or restoring work participation in workers with LBP and LRS.

## Methods

The guideline development process was guided by the Guideline Core Group (GCG), preparing the evidence tables and formulating the concept recommendations. In six meetings (October 2018–May 2020) the evidence tables and recommendations for the different topics of the guideline were discussed, refined and determined in a broader team, the Guideline Development Group (GDG), a multidisciplinary expert panel.

### Evidence Search in Literature

A broad search strategy using the search string of the guideline ‘Low back pain and sciatica’ of the National Institute for Health and Care Excellence [7] with the additional term ‘intervertebral disc displacement’ was performed. The search was limited to systematic reviews, additional search strings including terms for work and functioning were added. The literature search was conducted in Medline, EMBASE, and in the Cochrane Database of Systematic Reviews on 30 November 2018 (*Supplement 1 Online Resource*). The search resulted in a total of 1458 articles. Two reviewers (JWHL&JLH and JWHL&PPFMK) screened title and abstract of all papers. Systematic reviews in English, Dutch or German with the most recent evidence (from 2010-present) were included. Duplicates, studies off topic, studies with cohorts of patients with LBP and LRS within other patient populations or studies not describing relevant work-related outcomes were excluded. Deleting duplicates and selecting the period from 2010–2018 resulted in 758 articles, of which another 460 articles were removed due to the other exclusion criteria. Of 298 articles the full text versions were read, resulting in 208 exclusions and 90 inclusions (*Supplement 1 Online Resource*).

For some interventions insufficient or no evidence was found in the included reviews from the period 2010–2018. To fill this gap, evidence from other sources, like recent original studies, evidence-based guidelines and reviews prior to 2010, was added and evaluated.

### Grading of Evidence

One investigator (JWHL) extracted the following data sorted per research question: study characteristics, characteristics of the included populations, effect sizes and authors conclusions. For rating the certainty of the evidence we used the GRADE method (Grading of Recommendations Assessment, Development and Evaluation) [10, 11]. If GRADE was used in the included (Cochrane) systematic reviews, we adopted the rating. If this rating was absent, we assessed the certainty of the evidence ourselves, using the available data in the systematic reviews. For the prognostic factors in workers with LBP we selected one review [12]. The certainty of evidence in this study was assessed using the rating system of Hoogendoorn et al. [13], and we adopted these outcomes.

Separate evidence tables were created for the risk factors and the prognostic factors for both LBP and LRS, including the relevant variables: risk or prognostic factor, used systematic review, included studies, number of participants, number of cases/incidence, FU time, (pooled) effect variable, effect size including 95% CI, limitations according to GRADE: study quality, consistency, directness, precision and probability of publication bias and the resulting GRADE rating (high, moderate, low, very low). In the evidence tables for prevention of LBP/LRS and interventions for both LBP and LRS, the following variables were also included: comparison, prevention/intervention, work-related outcome, follow-up period (short term < 3 months; intermediate term > 3 months and < 12 months; long term ≥ 12 months; and very long term ≥ 24 months). The results were checked by one investigator (MWL), and discussed with all members of the core group (GCG).

### Formulating Guideline Recommendations

Concept recommendations were formulated by the core group and discussed in the broader team using the GRADE evidence to decision (EtD) framework [14], following the seven factors: (1) quality of evidence; (2) balance between benefits and harm; (3) values and preferences of the patient; (4) values and preferences of the professionals; (5) medical costs (resource allocation); (6) feasibility; and (7) acceptability and equity. Only interventions effective for work related outcomes were included in the recommendations.

The formulation of the recommendations generally followed the quality of evidence, using “*Advise*…” in case of high or moderate quality evidence / high or moderate certainty and started with “*Consider*…”, in case of low to very low-quality evidence / low to very low certainty. Exceptions to these rules were made when other factors, like patient wishes or expert experience or opinions were introduced by the GDG during the discussion about the recommendation. Formulations were then adapted, regardless of the certainty of the evidence. This also applies to recommendations based on opinion expertise.

### Layout Guideline

The recommendations in the guideline were presented as a pathway for OP’s, starting with a patient who has low back pain, going through the process of assessment, history taking and ongoing consultations and treatment. This includes a) Advice about interventions for recovery of daily functioning; (b) Prevention phase to asses risk factors and advice about prevention; c) Interventions to enhance work participation in case of (1) complaints, (2) sick leave, (3) persistent complaints after 6 weeks, and (4) after 12 weeks.

### External Independent Review

In order to perform an extra quality check and to ensure implementation in daily practice, the concept guideline with the final recommendations was send to experts in various fields: occupational physicians, insurance physicians in social medicine, insurance physicians in private medicine/medical officers, researchers, patients and related professional associations for general practitioners, occupational physiotherapists, physiotherapists, orthopaedic surgeons and neurologists. 42 Persons responded, comments were categorised, analysed and discussed in the development group, resulting in final adaptations of the guideline.

### Guideline Authorisation

The guideline was submitted to the authorisation committees of the three Dutch organisations for OH physicians: for occupational physicians (NVAB), for physicians in social insurance medicine (NVVG) and for medical officers in private insurances (GAV).

## Results

**Fig. 1:**
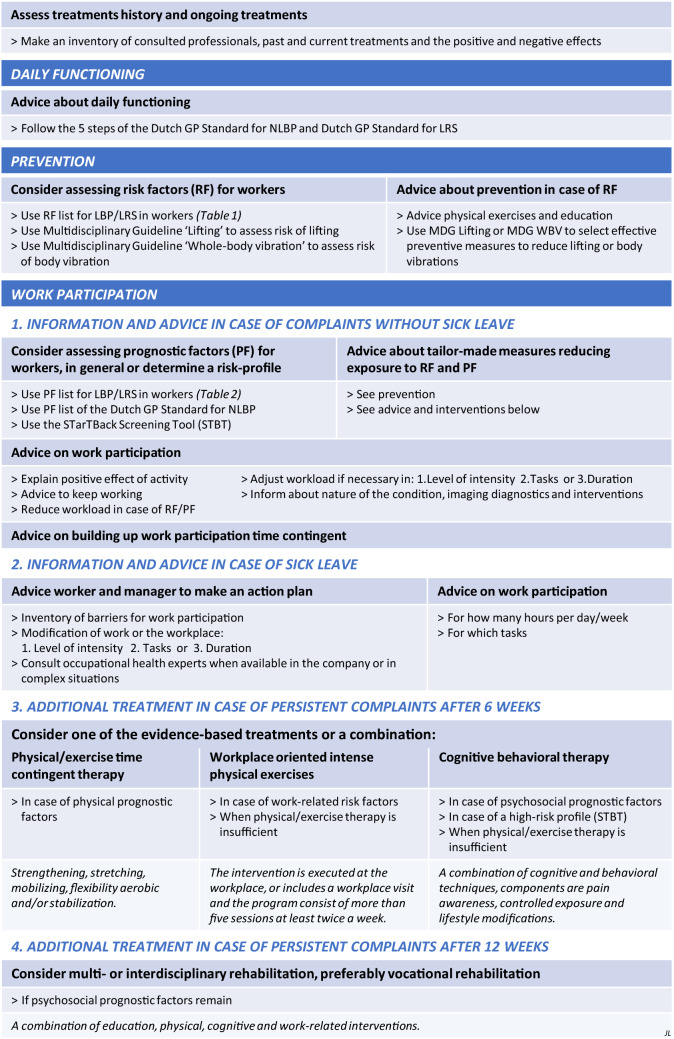
Intervention strategy of occupational health care professionals for workers with LBP or LRS

The main goal of the treatment policy in occupational health is to maintain or restore work participation, i.e. work ability, staying at work and return to work. Therefore the GDG formulated an intervention strategy (Fig. 1), divided into three sections: a) daily functioning; b) prevention; and c) work participation. The recommendations effective for work participation for workers with LBP and LRS (c), consists of four stages: (1) providing information and advice in case of complaints; (2) in case of sick leave; (3) considering additional treatment in case of persistent complaints (i.e. sick leave) after 6 weeks; and (4) after twelve weeks. The recommendations for work participation in case of existing LBP and LRS are preceded by recommendations about prevention of development of (work-related) LBP and LRS (b). Since advice on restoring daily functioning can also be relevant for occupational health care providers, an additional recommendation aiming at daily functioning was formulated (a). The intervention strategy starts, like in common practice, with an inventory of current and previous care.

In case the worker with long-term continuous complaints due to LRS decides, in consultation with his general practitioner and surgeon, for the option of surgery, the GDG formulated recommendations regarding pre- and postoperative advice to promote work participation.

### Recommendations for the Intake

*Ask the patient about current and previous care by other (para) medical professionals and its effects and consult these colleagues if their care might influence work participation*.

Based on expert advice, the GDG recommends an inventory of all completed and ongoing treatments by other (para) medical professionals and their effects on work participation to prevent overtreatment and ineffective care [15]. In addition, other treatment policies aimed at restoring daily functioning should also be considered.

### Recommendations for Daily Functioning

*Follow the guidelines for GPs on LBP and LRS that support interventions directed at pain and daily functioning*.

The GDG recommends education, guidance and measures following a stepped care approach: the most effective, yet least resource intensive treatment is delivered first. This advice is based on the evidence-based Dutch Standards for GPs for Nonspecific Low Back Pain [16] and for Lumbosacral Radicular Syndrome [17]. The guidance in the standards is composed of five steps. The first step is to inform the patient and advise him to keep moving despite pain and hindrance including referral to an OP in case of a (possible) relation with work or in case of sick leave. Step 2, initiated after about 3–4 weeks of complaints, is to promote building up activities conform a time contingent protocol, in case of avoidance behavior supported by a physiotherapist or exercise therapist. If complaints remain, the intensity of treatment is increased. Treatments in the next steps are: Step 3, exercise therapy after 6 weeks of complaints; Step 4, behavioural treatment after 12 weeks, followed by Step 5, multidisciplinary rehabilitation if complaints persist for a longer period. During this time, pharmacological interventions for pain relieve could support the activation strategy [16].

### Recommendations for Prevention

*Consider assessing risk factors for LBP and LRS for workers*.

Risk factors play an important role in the onset and development of a new episode of LBP and LRS or a recurrence and the complaints resulting from LBP and LRS like pain, disturbed functioning or influence on the workability resulting in reduced work participation or full/partial sick leave. Risk factors in workers differ slightly (Table 1) from the risk factors in the general population. OH professionals should consider an inventory of both work-related, as well as personal-related risk factors to inform workers and employers about specific measures related to work. This recommendation is based on very low-quality evidence for risk factors for LBP in a working population [18], and high to very low-quality evidence for risk factors for LRS in working population [18, 19].

**Table 1:** Risk factors for LBP and LRS in workers

Risk factors	LBP [18]	LRS [18, 19]
Work-related Physical	Flexed posture (> 45–60° trunk for > 5% of the time) Lifting (> 25 kg or repetitive 3–25 kg) Whole-body vibrations (driving 10-14 h p/w) Bending forward and backward (often) Pulling (> 25 kg), kneeling (> 15 min), standing (> 30 min/h) Working with hands above shoulders (> 15 min)	Lifting and bending of the trunk Heavy physically demanding work or manual laborer (> 2 h p/d) Working in kneeling or squatting position (> 1u p/d) Working with the trunk forward flexed (> 2 h/d) Bending and twisting of trunk Whole-body vibrations (driving > 2 h 1 × p/w) Lifting and carrying Working with hands above shoulders (> 1 h p/d)
Work-related Psychosocial	Highly monotonous work Low job security Low social support from coworkers and supervisor High job strain Low supervisor support High job demands Low job control	*No factors found in literature*
Personal Physical	*No factors found in literature*	*No factors found in literature*
Personal Psychosocial	Depression, mental distress- being stressed, nervous or tense Dissatisfaction with life Psychosomatic factors	*No factors found in literature*
Personal Lifestyle	Obesity (BMI > 30) Smoking	Smoking Overweight (BMI 25–29.9) and obesity (BMI > 30)
Risk groups	Age (< 45 year) in women Previous episode(s) of low back pain	Age (> 60 year) Height (> 1.80 m) Previous episode(s) of low back pain

*LBP* low back pain, *LRS* lumbosacral radicular syndrome

*If risk factors are present, consider preventive interventions and tailor-made measures to eliminate or reduce risk factors obstructing work participation*.

For two specified risk factors, ‘lifting’ and ‘whole-body vibration’, the GDG composed two separate recommendations based on the evidence from two practice guidelines. When lifting is a regular task at work, OH professionals should evaluate the risk using the evidence-based multidisciplinary practice guideline “Reduce the workload due to lifting for preventing work-related low back pain” [19]. This guideline supports occupational safety and health professionals in assessing the risk associated with lifting and selecting effective preventive measures for low back pain like eliminating manual lifting, improve lifting situations by optimizing working height and reducing load mass, and take organizational measures, for example composing lifting teams. When occupational exposure to whole-body vibration from the operation of vehicles is present, OH professionals should use the evidence-based practice guideline ‘Reducing exposure to body vibrations to prevent back problems’ [20], to assess the risk and to give advice to prevent or reduce the consequences of body vibrations when needed. This can be achieved through workplace-oriented technical and organizational interventions reducing vibrations, and individual instruction and exercises for the worker.

When other physical risk factors, like working with a flexed posture or with the hands above the shoulder, are present in work, OH professionals should advise physical exercises. This can be advised to prevent the development of LBP and LRS or to prevent recurrent LBP and LRS. Three reviews provided low-quality evidence of a protective effect of exercises on the occurrence or risk of LBP [21–23]. Very low-quality evidence was provided that exercise reduces the risk of sick leave due to LBP in the long-term [23] or resulted in less days of sick leave [21]. Despite the low to very low certainty of the evidence, the GDG decided to formulate this recommendation as an advice, based on expert opinion. Exercises should be tailor-made, i.e. related to present risk factors in work and the physical condition of workers. The focus is either on muscle strength and muscle endurance exercises of abdominal, leg and back muscles, stretching exercises of back, hip and leg muscles, balance exercises and functional coordination training, cardio training or a combination.

Exercises should optionally be performed under supervision of a professional and preferably be combined with education. This recommendation was based on moderate to low [23] and very low-quality evidence [22] for reduction of the risk of an episode of LBP at short-term follow-up. The GDG considered education, consisting of anatomy of the back, information about biomechanical principles, pathology and pain mechanisms, supportive to the explanation of the importance of staying active despite experiencing pain.

### Recommendations for Work Participation

The stepped care strategy was applied in the practice of OH professionals with interventions aiming at work participation. Executing stepped care, recommending the most effective, least resource intensive treatment first, demands that OH professionals not only take into account the duration and severity of the complaints. Additionally, the presence of risk factors and prognostic factors should be identified with prognostic tools in the first phase in case of complaints without sick leave (1). Knowledge of the presence of these factors results in earlier, more effective, stepped care referral and more specific counselling in the phase of persistent complaints after six (3) or 12 weeks (4).

OH professionals should also include effects of previous interventions, current treatments and motivation of the patient, i.e. treatment options should be discussed with the patient, based on the principles of shared decision making.

#### 1) Information and Advice in Case of Complaints Without Sick Leave

*Consider assessing prognostic factors for LBP and LRS for workers, consider assessing the general prognostic factors for LBP and LRS or determination of a risk profile of the psychosocial factors using a prognostic tool, such as the STarT Back Screening Tool*.

OH professionals should consider identification of factors influencing the progress of LBP and LRS in workers, the prognostic factors. We distinguished prognostic factors with a negative or positive effect, i.e. hindering or supporting work participation due to subacute and chronic LBP [12] and due to LRS [24] (Table 2), based on moderate to low-quality evidence. An inventory of the prognostic factors of LBP and LRS in the general population hindering daily functioning from the evidence-based Dutch GP’s Standard for NLBP [16] can also be considered. If psychological prognostic factors affect work participation, assessment of a risk profile can be considered, to enable stratified management [25]. The GDG suggests to use the STarT Back Screening Tool (SBST) [25], in line with physiotherapists and GPs [9]. This screening tool is a 9-item questionnaire generating an overall score and psychosocial sub-score that categorizes people into low, medium and high risk of persistent back pain-related disability [26, 27].

**Table 2:** Prognostic factors influencing work participation in workers with LBP and LRS

Prognostic factors	Negative effect		Positive effect	
	LBP [12]	LRS [24]	LBP [12]	LRS [24]
Work-related Physical	High physical job demands	Higher physical demands^1^	Lower physical demands	*No factors found in literature*
Work-related Psychosocial	*No factors found in literature*	*No factors found in literature*	*No factors found in literature*	*No factors found in literature*
Personal Physical	High pain intensity Small increase of functionality High physical demands in daily live	*No factors found in literature*	Low pain intensity Better general health status Good cardiovascular fitness (FCE) Improved trunk flexibility after training Less functional limitations	Lower pain intensity^2^ Negative SLR-test^2^ Less disability by LRS^2^ Higher physical function^2^
Personal Psychosocial	Low recovery expectations Pain catastrophising Inadequate coping Fear avoidance Low cognitive appraisal	More fear avoidance^1^	*No factors found in literature*	Less fear avoidance^2^
Risk Groups	Male sex Higher age	Older age^1^	Higher socio economic status (SES)	Younger age^1,2^

*LBP* low back pain, *LRS* lumbosacral radicular syndrome

^1^Factors found in a surgical population;

^2^Factors found in a mixed population, with surgical and/or conservative treatments

*If prognostic factors are present or in case of a high-risk profile on the SBST consider interventions to eliminate or reduce factors hindering work participation and to support the beneficial factors*.

In case of existing prognostic factors or a high-risk profile, OH professionals should advise interventions to overcome hindering factors for work participation like high physical job demands, or support the beneficial factors like improving a worker’s general health.

The primary aim for the management of LBP and LRS is the maintenance or restoration of work ability and enhance work participation. With information and advice, the OP provides workers with tools to participate in work despite the limitations caused by LBP and LRS.

*Inform and advise the worker and/or employer, if necessary, in consultation with other occupational health experts, about work participation despite having LBP and LRS using the following points*:

- *Advice about the positive effect of an active approach on pain and functioning.*
  Based on expert opinion [15], OH professionals should advise from the thought of the notion of positive health, by explaining people that active strategies are associated with reduced disability. OH professionals should provide workers with information on the nature of LBP and LRS, like back anatomy, biomechanical principals and pain mechanism, and encourage them to continue with normal activities, based on moderate to very-low quality evidence [25].
- *Advice to keep working as much as possible, despite pain.*
  Explain that participation in work supports recovery including reduction of pain and limitations. Pain is no reason to limit physical activity [28].
- *Advice to reduce workload caused by work-related risk factors and prognostic factors.*
  OH professionals should facilitate work participation by advising specific interventions to reduce present work-related risk factors and prognostic factors [7], such as eliminating manual lifting when lifting is a risk factor or explaining the positive effect of being active in case of fear avoidance behavior.
- *Advice to keep working as much as possible, if necessary, through temporary modification of workload, first in intensity, or else in tasks or duration.*

Based on moderate quality evidence [29] and expert opinion, OH professionals should advice to enable work participation through (short-term) modification of work environments such as adaptation of the level of intensity of the physical demands, or else in tasks or duration of the physical demands.

*Advice to increase work participation according to a time contingent approach*.

OH professionals should advise building up activities on time rather than based on pain to reduce fear avoidance behavior, analogous to the second step of the evidence-based Dutch GP’s Standard for NLBP [16].

#### 2) Information and Advice in Case of Sick Leave

*Advice to make an action plan facilitating return to work as soon as possible in case of sick leave*.

OH professionals should advise the worker and supervisor to make an action plan in case of sick leave, based on moderate quality evidence [30]. Experts in the field of work and health, like occupational hygienists, physiotherapists, nurses and labour experts can be consulted when available in the company or in case of complex situations (opinion GDG), the OP has a coordinating role. An inventory of barriers and facilitators for work participation is advised as well as modification of work or the workplace to facilitate return to work, preferably describing the level of intensity of back straining work, and the kind of tasks or duration (expert opinion).

*Advice about return to work, defining tasks and hours*.

Advice on staying at work or return to work in case of sick leave, by defining for how many hours and for which tasks work can be resumed. The aim of a gradual RTW plan is to build up work participation in the next weeks.

In line with the stepped care strategy, OH professionals should consider additional interventions, when the above-mentioned interventions are not adequate to restore work participation for the number of hours and or tasks as advised. Based on experience and opinion of the GDG and in line with the evidence-based Dutch GP’s Standard NLBP [16], this step should be considered after about 6 weeks of complaints. Table 3 presents the evidence for the additional interventions including the limitations, i.e. the downgrade factors, and GRADE rating.

**Table 3:** Summary of evidence effective interventions on work for LBP and LRS with GRADE rating

Intervention^1^	Comparison^1^	State LBP^2^	Outcome^3^	FU^4^	Systematic review	No st^5^	Studies	N^6^	Effect	Effect Size^7^	Lower limit 95% CI	Upper limit 95% CI	Limitations^8^	GRADE^9^
Physical exercise	Unknown	Unknown	Work disability	LT	Oesch 2010	8	Hagen 2000 Karjalainen 2003 Lindström 1992 Niemistö 2003 Skouen 2002 Staal 2005 Steenstra 2006 Torstensen 1998	1992	OR	0.66	0.48	0.92	*Inconsistency*	*Low*
IPCP	CaU	Chronic	Time to RTW	ST (3 m)	Schaafsma 2013	1	Bendix 1996	74	OR	0.16	0.05	0.49	Very serious risk of bias Imprecision	Very low
IPCP	CaU	Chronic	Time to RTW	LT (12 m)	Schaafsma 2013	5	Mitchell 1994 Bendix 1996 Corey 1996 Jensen 2001 Skouen 2002	1039	SMD	− 0.23^$^	-0.42	-0.03	Serious risk of bias	Moderate
IPCP + CaU	CaU	Chronic	Time to RTW	LT (12 m)	Schaafsma 2013	1	Lambeek 2010	134	SMD	− 4.42	-5.06	-3.79	Imprecision	Low
IPCP + CaU	CaU	Subacute	Time to RTW	VLT (> 24 m)	Schaafsma 2013	2	Staal 2004 Lindstrom 1992	237	SMD	− 0.39	-0.76	-0.02	Imprecision	Moderate
IPCP	ET	Chronic	Time to RTW	VLT (> 24 m)	Schaafsma 2013	1	Bendix 1997	52	SMD	− 0.62	-1.21	-0.04	Very serious risk of bias Imprecision	Very low
RTWI	CaU	Subacute	RTW	IT (6 m)	Hlobil 2005	6	Hagen 2000 Staal 2004 Rossignol 2000 Indahl 1998 Lindström 1992 Loisel 1997	1773	Significant positive effect				*Serious risk of bias*	*Moderate*
RTWI	CaU	Chronic	Days of sick leave	LT (12 m)	Hlobil 2005	3	Gatchel 2003 Hagen 2000 Staal 2004	715	Significant positive effect					*High*
RTWI	CaU	Chronic	Days of sick leave	VLT (24 m)	Hlobil 2005	1	Lindström 1992	103	Significant positive effect				*Serious risk of bias* *Imprecision*	*Low*
RTWI	CaU	Chronic	Days of sick leave	VVLT (36 m)	Hlobil 2005	1	Hagen 2000	457	Significant positive effect					*High*
WPI	CaU	Chronic	First sick leave period	LT (12 m)	Van Vlisteren 2015	2	Lambeek 2010 Anema 2007	330	HR	1.77	1.37	2.29	*Imprecision*	*Moderate*
CI	No	Acute*	RTW < 3 m	VLT (24 m)	RCT**	1	Nicholas 2019	109	OR	0.26	0.07	0.98	Indirectness Imprecision	Low
MBR	CaU	Chronic	RTW	LT (12 m)	Marin 2017	3	Bultmann 2009 Loisel 1997 Whitfill 2010	170	OR	3.19	1.46	6.98	Very serious risk of bias Imprecision	Very low
MBR	CaU	Chronic	Sick leave days	LT (12 m)	Marin 2017	2	Karjalainen 2003 Schiltenwolf 2006	210	SMD	− 0.38	− 0.66	− 0.10	Serious risk of bias Imprecision	Low
MBR	PT	Chronic	Proportion working	IT (3-12 m)	Kamper 2014	3	Bendix 1996/1998 Henchoz 2010 Jousset 2004	221	OR	2.14	1.12	4.10	Serious risk of bias Imprecision	Low
MBR	PT	Chronic	Proportion working	LT (≥ 12 m)	Kamper 2014	8	Alaranta 1994 Bendix 1996/1998 Bendix 2000 Henchoz 2010 Kapaa 2006 Kool 2007 Roche 2007/2011 Streibelt 2009	1006	OR	1.87	1.39	2.53	Serious risk of bias	Moderate
MBR	ACG	Chronic	Proportion working	IT (3-12 m)	Hoffman 2007	3	Bendix 1998 Christensen 2003 Brox 2003	245	ESD	0.36	0.06	0.65	*Imprecision*	*Moderate*
MBR	ACG	Chronic	Proportion working	LT (≥ 12 m)	Hoffman 2007	4	Alaranta 1994 Bendix 1998 Christensen 2003 Corey 1996	609	ESD	0.53	0.19	0.86	*Inconsistency*	*Moderate*

^1^Interventions and comparisons: *IPCP* Intense Physical Conditioning Programme, *CaU* Care as Usual, *MET* Multidisciplinary exercise treatment, RTWI Return-To-Work Intervention, with physical exercise or advice about it and education in all the interventions, behavioral treatment (N = 6), ergonomic measures (N = 2) and case management (N = 6). *WPI* Workplace Intervention based on participatory ergonomics including integrated care management with/without graded activity programme (time contingent programme based on cognitive behavioral principals). *CI* Cognitive Intervention. *MBR* Multidisciplinary Biopsychosocial Rehabilitation. *PT* Physical Therapy. *ACG *active control group. *LBP* low back pain. *LRS* lumbosacral radicular syndrome

^2^State LBP: Acute back pain: Duration of symptoms less than 6 weeks. Subacute back pain: Duration of symptoms more than six but less than 12 weeks. Chronic back pain: Duration of symptoms more than 12 weeks

^3^Outcome: RTW: Return to work

^4^FU: *ST* Short term < 3 m. *IT* Intermediate term > 3 m and < 12 m. *LT* Long term ≥ 12 m. *VLT* Very long term ≥ 24 m

^5^No st Number of studies

^6^ N Number of participants

^7^Effect sizes Are significant

^8^Limitations: Limitations adopted from review or *limitations determined by the authors of this article on the basis of limitations found in the review or original studies* (in italic)

^9^GRADE qualifications: GRADE qualifications adopted from review or *limitations determined by the authors of this article* (in italic)

^$^Statistically significant, not clinically relevant

^*^Patient group: soft tissue injury and high risk at the Örebro Musculoskeletal Pain Screening Questionnaire–short form (ÖMPSQ-SF)

^*^RCT, introduced by a GDG-member to determine effect of Cognitive Intervention and the GRADE qualification, in case of a shortage of relevant reviews

#### 3) Additional Treatment in Case of Persistent Complaints (e.g. Sick Leave) After 6 Weeks

*Consider advising about additional physical, workplace-oriented or cognitive treatment in case of persistent sick leave due to LBP and LRS after about 6 weeks*.

If information and advice does not improve work participation, OH professionals should recommend additional interventions. The choice is, besides the previously mentioned factors, based on prognostic factors: Physiotherapy or exercise therapy in case of physical factors or as a first step; a workplace-oriented physical program in case of work-related physical factors; and a cognitive behavioural intervention if psychosocial factors are present.

*Consider physiotherapy or exercise therapy with a time-contingent approach*.

OH professionals should consider physiotherapy or exercise therapy after about 6 weeks if relevant physical work- or person related prognostic factors are present, based on low-quality evidence in eight RCT studies [31]. The studied physical exercises encompass a wide variety of interventions aiming at strengthening, stabilization, stretching, mobilizing, flexibility and/or aerobic capacity applied with different rationales: individual/standard designed, home/supervised, high/low-dosed, in/outpatient, work/not-work related, with/without behavioural treatment approach. Since meta-regression analysis showed no significant differences between the various exercises, the OH professional should advise exercises tailored to the patient.

*Consider high-intensity workplace-oriented physical conditioning*.

When general physical or exercise therapy is insufficient to restore work participation, OH professionals should consider a more intense physical conditioning program (IPCP), based on very low quality at the very long term. Such a program consists of more than five sessions at least twice a week [32]. When a work-related physical risk factor or prognostic factor is present, a workplace intervention should be considered based on high to moderate quality evidence.

Based on moderate quality evidence on long-term outcomes in five RCT’s [32], the GDG recommends a work-related intense physical conditioning program.

*Consider cognitive behavioural intervention*.

Cognitive behavioural therapy involves a combination of cognitive and behavioural techniques, aiming to identify, challenge, and subsequently change patterns of unhelpful thoughts, beliefs, attitudes and behaviours. Components are pain awareness, controlled exposure and lifestyle modifications.

Based on low-quality evidence in a recent RCT [33], OH professionals should consider a cognitive behavioural intervention, when relevant psychosocial risk or prognostic factors are present, in case of a high-risk profile scored on the STarT Back Screening Tool, or when (work-related) physiotherapy or exercise therapy did not result in (full) work participation.

If monodisciplinary treatments are insufficient to achieve work participation within about 12 weeks (experience and opinion GDG), OH professionals should consider the assignment to a rehabilitation program involving multiple disciplines.

#### 4) Additional Treatment in Case of Persistent Complaints (e.g. Sick leave) After 12 Weeks

*Consider multi- or interdisciplinary rehabilitation, preferably vocational rehabilitation if full return to work is not completed after about 12 weeks*.

OH professionals should consider a multi- or interdisciplinary rehabilitation program, based on moderate to very low-quality evidence [34–36]. Vocational rehabilitation is preferred (expert opinion). Multidisciplinary rehabilitation combines education, physical, cognitive-behavioural and work-related interventions. This step should only be considered if (full) work participation is not achieved despite treatment for a period of about 12 weeks, especially if psychosocial components remain.

### Recommendations in Case of Surgery LRS

The Dutch Standard for LRS, recommends that general practitioners discuss the possibility of surgery with the patient, when strong radiating pain from LRS remains after six till eight weeks [17]. Based on their experience and opinion, the OP should check this process with the patient and, if necessary, consult the GP. The GDG defined specific recommendations that could assist the OP in his additional role to discuss consequences for work participation in case of surgery.

*Advise the patient with LRS in case of surgery about the consequences for work participation and the associated treatment process*.

In case surgery is considered, the OP should discuss the pros and cons for conservative and operative intervention in relation to the consequences for work participation, like temporary sick leave and modification of work. OPs should also discuss the likely course and recovery and the action plan to be followed for return to work, including intervention strategies like graded activity.

During the postoperative process the focus is on information and stimulation of expanding activities.

*Inform and advise postoperatively about the expected course of recovery regarding work, stimulate activities and building up work participation, if necessary, in consultation with the general practitioner or surgeon*.

After surgery, the OP supports the patient to return to work, through information about the expected course, stimulation to expand activities in daily live, like personal care and walking, as well as building up work participation, with increasing workload (load, tasks, time). Discuss with the patient about the advice of the surgeon and contact surgeon or GP in case of doubts about work participation.

If work participation after 6 weeks is insufficient, the GDG recommends additional treatment to support recovery.

*Advice postoperative exercise therapy, preferably high intensity training guided by a physiotherapist or exercise therapist, if work participation is insufficient after 6 weeks*.

OH professionals should advice an intensive exercise program consisting of at least three supervised sessions a week, based on low-quality evidence for exercise therapy of two RCT studies and low-quality evidence for intensive exercise therapy of one RCT [24]. The effective exercises focused on intensive muscle strengthening [21] and on intensive supervised exercises for relaxation, stretching and stabilization [37].

## Discussion

There is a need for evidence that focuses on work related outcomes in the management of workers with LBP and LRS. The present guideline is the first guideline for occupational health care professionals with the focus on evidence-based interventions to enhance work participation in a working population. The American College of Occupational and Environmental Medicine’s Low Back Disorders Guideline [38], aiming at the management of low back disorders among working-age adults, also included recommendations about work activities. However, the reviewed interventions evaluated outcomes mainly focusing on pain and daily activities. Studies with work-related outcomes, like return to work and number of sick leave days, are only part of the whole evaluation, and not mentioned separately. The same applies to the previous guideline of the Netherlands Society of Occupational Medicine [39]. Notably, between the numerous clinical guidelines for the management of low back pain that have been published recently [7–9, 16, 17, 25, 38, 40], this guideline distinguishes itself from the rest through its focus. With recommendations effective for work participation based on an up-to-date evidence-based review of interventions, it is a relevant addition to the existing guidelines for occupational health care providers.

### Innovations

This guideline takes full advantage of the position of the OH professional at the workplace. The recommendations are aimed at work participation and structured as stepped care. Prevention at the workplace is included, starting with a risk assessment. Information about work participation and work-related advice are prominent in the first and second step of the intervention phase, which is preceded by a prognostic assessment. Only when these work-related interventions are not sufficient to restore work participation, additional interventions should be considered.

Furthermore, the recommendations of the previous occupational guideline [39] were mostly based on evidence of effective interventions for daily functioning and pain. The present guideline adds evidence on intervention strategies enhancing work participation and complements existing recommendations to improve daily functioning. The description of risk factors and prognostic factors is more detailed and also specified for workers, as well as interventions for the prevention of LBP and LRS. Furthermore, the classification of aspecific, acute and chronic low back pain and linked intervention strategy has been replaced by a stepped care strategy based on multiple components, like duration and severity of the complaints and the presence of risk factors and prognostic factors. Moreover, this guideline includes recommendations for both occupational and insurance physicians, as well as recommendations for cooperation between both professions and communication with other medical and OH professionals.

### Strengths

The strength of this guideline development process is the updated systematic approach resulting in traceable evidence-based recommendations. GRADE was not only used for a systematic rating of the quality of evidence, but the formulation of the recommendations resulted from the application of the systematic structure of the GRADE evidence to decision framework [10, 11].

The guideline process was aimed at optimal acceptance and implementation in occupational health practice. To reach this goal various steps were included in the development process. Guideline topics were determined in consultation with stakeholders relevant to the occupational field. Beside the evidence and the opinions of experts, the experience and opinions of medical specialists and patients played an important role in the formulation of recommendations. Consulting field experts, like OH professionals, researchers, medical professional associations and patients, who assessed the guideline and proposed suggestions for improvement, provided an extra quality step, and also improved the connection with daily practice.

The implementation process will be promoted by the cooperation with the professional occupational health organisations from occupational physicians, insurance physicians and medical officers. Next to authorisation of the guideline, these organisations are also involved in dissemination of the guideline among their members, with promotion of its release, distribution of the digital version, publication on their websites and training.

Another strength of the current guideline is the connection to the treatment policy of other (para)medical professionals. This is, for example, addressed in the evidence to decision process and in the referral to the treatment policy for pain and daily functioning in primary care. This connection prevents an isolated intervention strategy from the occupational health care providers, ignoring treatment by other (para)medical professionals.

### Limitations

Besides the strengths, we faced a limitation, by choosing systematic reviews as inclusion criteria in the literature search. The recommendations about interventions effective for work participation are restricted to those described in the included SR’s. However, systematic reviews do not comprise the most recent knowledge. For some interventions, like cognitive behavioural therapy, this gap could be compensated consulting an original study with updated knowledge from a recent RCT [33]. For the effect of spinal manipulative therapy on work participation however, this information was not available at the time of guideline development [41]. Another limitation was that for the guidelines methods we adopted systematic review methodology as proposed by PRISMA, but could not comply with all the requirements like data-extraction by 2 authors, or publication of the SR protocol.

### Recommendations for the Future

Inclusion of work-related outcomes is recommended for future studies in the area of effective interventions for LBP and LRS. To date, evidence for the effectiveness of several treatments on return to work is not sufficient, in contrast to the evidence for pain and daily functioning. Due to the absence of work-related outcomes, it was not possible to formulate recommendations enhancing work participation for some treatments, for example for spinal manipulation.

The lack of cost-effectiveness studies for interventions with work-related outcomes resulted in a modest role for the factor ‘medical costs’ (resource allocation) in the evidence to the decision process of this guideline. This suggests even more challenges for future research in this area [15, 42].

## Conclusion

Based on a systematic review process, a multidisciplinary occupational health guideline to enhance work participation among workers with LBP and LRS has been developed. This guideline is a supplement to existing clinical guidelines. By adding specific recommendations regarding work participation to the recommendations on recovery of daily functioning, the management strategy for workers is complete. Key points in the guidance of workers with LBP or LRS are the inventory of risk and prognostic factors, including effective prevention interventions for workers, and facilitating work participation through information, advice and, if necessary, considering additional interventions. This guideline is therefore not only valuable to OH professionals, but as well as to other health care professionals like general practitioners and physiotherapists.

## Supplementary Information

Below is the link to the electronic supplementary material.

Supplementary file1 (DOCX 24 kb)

## Data Availability

All data is available through the corresponding author.

## Footnotes

1 Workers: People with paid work, without distinction according to type of employment contract (i.e. people without a contract of employment). The potential workers are those who do not work but can work or (probably) be able to work in the future. For example, job seekers or people who are looking for (paid or not) work after treatment and/or re-inspection.
