# Supplementary material for: The Dutch Multidisciplinary Occupational Health Guideline to Enhance Work Participation Among Low Back Pain and Lumbosacral Radicular Syndrome Patients
Source: J Occup Rehabil. 2021 Jul 27;32(3):337–52. doi: 10.1007/s10926-021-09993-4 (PMC9576671; doi:10.1007/s10926-021-09993-4)
Supplement: Supplementary file 1 — Supplementary file1 (DOCX 24 kb) [file 10926_2021_9993_MOESM1_ESM.docx]

**Literature Search (30-11-2018)**

Medline (OVID)

low back pain/

sciatica/

radiculopathy/

((lumbar or lumbosacral or lumbo-sacral or back) adj5 (pain* or ache* or aching)).ti,ab,kf

(backache* or lumbago or sciatica or sciatic neuralgia).ti,ab,kf

(radiculopathy or radiculitis or radicular pain*).ti,ab,kf

(nerve root* adj5 (pain* or avulsion or compress* or disorder* or pinch* or inflam* or imping* or irritat* or entrap* or trap*)).ti,ab,kf

or/6-7

(back* or lumbosacral or lumbo-sacral or lumbar).ti,ab,kf

8 and 9

or/1-5,10

(worka* or worke* or workg* or worki* or workl* or workp* or work capacity or work disabilit* or work abilit* or at work or work exposure or work related or work participation or workers or job* or employee or staff or personnel or occupation or occupations or occupational or outdoor work* or day shift* or night shift* or shift work* or vocational rehabilitation or sick leave or absenteeism or sickness absen* or absente* or presente* or "return to work" or vocational reintegration or retirement or pension or employment or unemployed or unemployment or work status or industries or industrial sector or volunteer* or voluntary worker* or repetitive work).ab,hw,kf,ti.

meta-analysis/

meta-analysis as topic/

(meta analy* or metanaly* or metaanaly*).ti,ab,kf

((systematic* or evidence*) adj3 (review* or overview*)).ti,ab,kf

(reference list* or bibliograph* or hand search* or manual search* or relevant journals).ab.

(search strategy or search criteria or systematic search or study selection or data extraction).ab.

(search* adj4 literature).ab.

(medline or pubmed or cochrane or embase or psychlit or psyclit or psychinfo or psycinfo or cinahl or science citation index or bids or cancerlit).ab.

cochrane.jw.

systematic review.pt

or/13-22

and/11,12,23

EMBASE (OVID)

low back pain/

sciatica/

radiculopathy/

radicular pain/

radiculitis/

exp "nerve root injury"/

(backache* or lumbago or sciatica or sciatic neuralgia).ti,ab,kw

((lumbar or lumbosacral or lumbo-sacral or back) adj5 (pain* or ache* or aching)).ti,ab,kw

(radiculopathy or radiculitis or radicular pain*).ti,ab,kw

(nerve root* adj5 (pain* or avulsion or compress* or disorder* or pinch* or inflam* or imping* or irritat* or entrap* or trap*)).ti,ab,kw

or/9-10

(back* or lumbosacral or lumbo-sacral or lumbar).ti,ab,kw

11 and 12

or/1-8,13

(worka* or worke* or workg* or worki* or workl* or workp* or work capacity or work disabilit* or work abilit* or at work or work exposure or work related or work participation or workers or job* or employee or staff or personnel or occupation or occupations or occupational or outdoor work* or day shift* or night shift* or shift work* or vocational rehabilitation or sick leave or absenteeism or sickness absen* or absente* or presente* or "return to work" or vocational reintegration or retirement or pension or employment or unemployed or unemployment or work status or industries or industrial sector or volunteer* or voluntary worker* or repetitive work).ab,hw,kw,ti.

systematic review/

meta-analysis/

(meta analy* or metanaly* or metaanaly*).ti,ab,kw

((systematic or evidence) adj2 (review* or overview*)).ti,ab,kw

(reference list* or bibliograph* or hand search* or manual search* or relevant journals).ab.

(search strategy or search criteria or systematic search or study selection or data extraction).ab.

(search* adj4 literature).ab.

(medline or pubmed or cochrane or embase or psychlit or psyclit or psychinfo or psycinfo or cinahl or science citation index or bids or cancerlit).ab.

((pool* or combined) adj2 (data or trials or studies or results)).ab.

cochrane.jx.

or/16-25

and/14,15,26

**Results Literature Search**

Records identified through database searching

N=1458

Records identified through database searching

N=758

Full-text articles assessed

for eligibility

N=293

Records included in qualitative synthesis

N=90

Records excluded

duplicates / language restriction / <2012

N=700

Records excluded

based on title and abstract

N=465

Full-text articles excluded

N=203
